# *NOTCH1* mutations influence survival in chronic lymphocytic leukemia patients

**DOI:** 10.1186/1471-2407-13-274

**Published:** 2013-06-04

**Authors:** Kerstin Willander, Ravi Kumar Dutta, Jonas Ungerbäck, Rebeqa Gunnarsson, Gunnar Juliusson, Mats Fredrikson, Mats Linderholm, Peter Söderkvist

**Affiliations:** 1 Department of Clinical and Experimental Medicine, Department of Hematology, County Council of Östergötland, Linköping University, SE-58185 Linköping, Sweden; 2 Department of Clinical and Experimental Medicine, Linköping University, Linköping, Sweden; 3 Department of Laboratory Medicine, Stem Cell Center, Hematology and Transplantation, Lund University, Lund, Sweden; 4 Department of palliative care, Stockholms Sjukhem, Stockholm, Sweden

**Keywords:** Chronic lymphocytic leukemia, *NOTCH1* mutations, *TP53* mutations, Prognostic markers

## Abstract

**Background:**

*NOTCH1* PEST domain mutations in chronic lymphocytic leukemia have recently been shown to be of prognostic relevance. Both NOTCH1 and NOTCH2 are constitutively activated in B-cell CLL but not expressed in normal B cells and may be involved in survival and resistance to apoptosis in CLL. We screened for mutations in different parts of both *NOTCH1* and *NOTCH2* genes and related the changes to survival and other known risk factors.

**Methods:**

In a cohort of 209 CLL patients, we used single strand conformation analysis to determine which of the samples carrying the *NOTCH* mutations and direct dideoxy sequencing was used to determine the exact nucleotide changes. Kaplan-Meier curves and log rank test were used to determine overall survival for *NOTCH1* mutated cases and Cox regression analysis was used to calculate hazardous ratios.

**Results:**

In the present study, we found *NOTCH1* PEST domain mutations in 6.7% of the cases. A shorter overall survival was found in patients with *NOTCH1* mutations compared to wildtype (p = 0.049). Further, we also examined the extracellular and the heterodimerisation domains of the *NOTCH1* gene and the PEST domain and heterodimerisation domain of the *NOTCH2* gene, but no mutations were found in these regions. *NOTCH1* mutations were most commonly observed in patients with unmutated IGHV gene (10/14), and associated with a more aggressive disease course. In addition, *NOTCH1* mutations were almost mutually exclusive with *TP53* mutations. In the combined group of *NOTCH1* (6.7%) or *TP53* (6.2%) mutations, a significant difference in overall survival compared to the wildtype *NOTCH1* and *TP53* was found (p = 0.002).

**Conclusions:**

Both *NOTCH1* and *TP53* mutations seem to be independent predictive markers for worse outcome in CLL-patients and this study emphasizes the contention that *NOTCH1* mutations is a novel risk marker.

## Background

Chronic lymphocytic leukemia (CLL) is a heterogeneous disease with variable clinical course characterized by a monoclonal progressive accumulation of mature CD5+ B-lymphocytes avoiding apoptosis. Some patients with an indolent disease need no or little treatment while others have a more adverse disease at diagnosis. No common genetic lesion, which causes the disease, has been found [1], but recurrent mutations in CLL involve *TP53* and *ATM*, and novel mutations in the *NOTCH1, SF3B1, MYD88, BIRC3* and *FBXW7* genes have been identified through next generation sequencing [2]. The CLL cases may be divided in two major groups regarding to mutated (M) or unmutated (UM) immunoglobulin heavy chain variable region gene (IGHV) where patients with an unmutated IGHV clone have a more adverse prognosis than patients with mutated IGHV gene [3,4]. By the means of FISH analysis, different chromosomal aberrations as deletion in 11q, 13q, 17p or trisomy 12 are found in about 80% of tumor cells in the CLL-patients [5].

Recently, *NOTCH1* mutations were found to be predictor of poor prognosis in CLL [6-11]. Furthermore a study of Rosati et al. [12] showed that NOTCH1 and NOTCH2, together with their ligands Jagged1 –and 2 are constitutively activated in B-CLL cells but not in normal B cells, suggesting that NOTCH signaling is involved in survival and resistance to apoptosis in CLL.

The NOTCH receptor is a membrane bound protein that consists of an extracellular, transmembrane and intracellular domain that can be released upon ligand interaction and transactivate target genes. The NOTCH signal pathway is activated by a ligand on a neighboring cell and plays an essential role in controlling proliferation, differentiation and survival. Following the receptor-ligand binding, the NOTCH receptor first undergoes a S2 proteolytic cleavage by ADAM proteinase in the extracellular domain, which then is followed by a S3 cleavage by a γ-secretase complex in the transmembrane domain releasing the intracellular NOTCH domain that translocates to the nucleus where it interacts with a transcription complex and acts as a transcriptional activator for multiple target genes [13]. The C-terminal part of the intracellular domain consists of a PEST region that is important for proteasomal degradation of the NOTCH receptor by binding to FBXW7, an E3 ubiquitin ligase, to limit duration of the NOTCH activity. A CT deletion in the C-terminal region results in removal of the PEST domain, a truncated NOTCH protein, and impaired NOTCH degradation and constitutive transcriptional activation of NOTCH target genes in CLL [7,14].

In the present study we have screened for mutations in different parts of both the *NOTCH1* and *NOTCH2* gene in a cohort of 209 CLL-patients. There is a high structural similarity between *NOTCH1* and *NOTCH2* genes and recent *NOTCH2* gain-of-function mutations are found in B-cell lymphomas [15]. Further, as NOTCH2 is involved in overexpression of CD23, one of the hallmarks of CLL [16], it prompted us to screen both the *NOTCH1* and *NOTCH2* genes for genetic alterations. Mutations were only found in the PEST region in the *NOTCH1* gene in our cohort and emerged as an independent factor of poor overall survival and disease stage, in addition to *TP53* mutations and IGHV gene status.

## Methods

### Patients

In this study, peripheral blood from 209 CLL patients (145 men and 64 women) was collected between 1996 and 2006 at the Department of Hematology, Linköping University Hospital. Mononuclear cells were isolated by Ficoll-Paque gradient centrifugation and genomic DNA was extracted by proteinase K digestion and stored frozen until used as earlier described [17]. The samples were collected either at the time of diagnosis or prior to the first treatment. For all patients, follow-up data were available, and for 106 live patients the median follow-up time was 6.8 years (range 1.6-14.9 years). The median age at diagnosis was 62.5 years (range 38.3-87.0 years). The immunophenotype and the Binet staging system were according to the IWCLL guidelines [18]. The immunoglobulin heavy chain variable region genes (IGHV) and *TP53* gene status were analysed and reported in an earlier study [17]. Informed consent was obtained from the patients and the study was approved by the regional ethical committee (Dnr 02–459) in Linköping and conducted in accordance with the ethical guidelines of the Helsinki Declaration.

### *NOTCH* mutation status detection

The *NOTCH1* mutations status were analyzed for the extracellular region (exon 6, 7, 8, 11, 12 and 13), the heterodimerisation domain (exon 26, 27) and the PEST region (exon 34) and the *NOTCH2* was only analyzed for mutations in the heterodimerisation and the PEST domains by PCR amplification followed by single strand conformation analysis (SSCA) according to the original protocol [19] and direct dideoxy sequencing to determine the exact nucleotide change and compared to corresponding *NOTCH1* and *NOTCH2* germline sequence (NM_017617.3 and NM_024408.3 respectively). Primer sequences are shown in Table 1.

**Table 1:** Primer sequences

**Gene-exon-segment**	**Application**	**Forward primer**	**Reverse primer**	**Product size/bp**	**T**_**A**_**/°C**
Notch1- 26-a	PCR/SSCA	AGCCCCCTGTACGACCAGTA	CTTGCGCAGCTCCTCCTC	283	63.5
Notch1- 26-b	PCR/SSCA	ACACGGCCAGCAGATGAT	GAGAGTTGCGGGGATTGAC	231	57.1
Notch1- 27	PCR/SSCA	GTGGCGTCATGGGCCTCA	TAGCAACTGGCACAAACAGC	342	63.2
Notch1- 34a	PCR/SSCA	AACCACCTGCCTGGGATG	CGCATTGACCATTCAAACTG	232	57.1
Notch1- 34b	PCR/SSCA	GGGCCCTGAATTTCACTGT	AGGCCCTGGTAGCTCATCAT	229	60
Notch1- 34c	PCR/SSCA	GCTGCACAGTAGCCTTGCT	CTGAGCTCACGCCAAGGT	224	58
Notch1- 34d	PCR/SSCA	ACATCCAGCAGCAGCAAAG	GTGGGACCAGCGAGGATG	222	58
Notch1- 34e	PCR/SSCA	CACTATTCTGCCCCAGGAGA	CAGTCGGAGACGTTGGAATG	234	58
Notch1- 34f	PCR/SSCA	ACAGCTACTCCTCGCCTGTG	AAGGCTTGGGAAAGGAAGC	248	58
Notch1-6	PCR/SSCA	GCAGCTGCCCGGGGCCGACA	TCAGGCCTGGCCCATGTGA	330	62
Notch1-7	PCR/SSCA	ATGCCTGGCCAGGGGCCGT	TCGACTTCTCATCGGTTCT	273	58
Notch1-8	PCR/SSCA	CCGATGGGGGTGGTGTGCAGT	TGCCCAGCCTCGACTCGGTT	331	63
Notch1-11	PCR/SSCA	AGTCCTAAGTCTTCCTGTGCC	AGGCCCGCCCTGCCCACT	325	65
Notch1-12	PCR/SSCA	AGGACTGACCGACACGTG	TCTGAGCACAGTGCAGTCA	183	53
Notch1-13	PCR/SSCA	TGGGCGCTGGGCCTCGGA	ACTGATGTGTCCCCATGA	268	54
**Gene-exon-segment**	**Application**	**Forward primer**	**Reverse primer**	**Product size/bp**	**T**_**A**_**/°C**
Notch2- 26-1	PCR/SSCA	TTCTCTGCTTCCCCTTACCT	TTAATGCGCAGGTTGGTGT	250	54.1
Notch2- 26-2	PCR/SSCA	TGGTATTGATGCCACCTGAA	GCCTTGAAGTTCAGAAACCAA	240	54.1
Notch2- 27	PCR/SSCA	TACCCCCATCTCTCCTCCTC	AATTGTTCCCCCAATTGACA	250	55.2
Notch2- 34-1	PCR/SSCA	TCCCCTGTTGATTCCCTAGA	CACAATGTGGTGGTGGGATA	249	55.2
Notch2- 34-2	PCR/SSCA	GCACTGTGCTTCCCTCAGT	CTGCCTTTAGGGATGAGCTG	298	55.2
Notch2- 34-3	PCR/SSCA	ACCCATCCTGGCATAGCTC	TAGGCTGGGAGAATGGTCTG	287	55.2
Notch2- 34-4	PCR/SSCA	TTTGCCCAGTGTGGCTTT	GGTGATGAACTTGACCACTG	249	57.1
Notch2- 34-5	PCR/SSCA	ACACCCAGTCACAGTGGTCA	TGTCTCTACACTGGAGGTGGAC	242	61.6

### *TP53* and IGHV gene status detection

*TP53* gene mutation analysis was performed for exons 5–8 (the DNA binding domains) by the PCR-single strand conformation analysis (SSCA) technique and samples displaying mobility shifts were sequenced with the dideoxy termination method to confirm the nucleotide changes.

The IGHV gene mutational status was performed by PCR amplification on genomic DNA by using specific VH/JH primers [20], followed by DNA sequencing of both forward and reverse strands. To determine the IGHV gene identity the sequences were aligned by using the IMGT/V-QUEST database (http://imgt.org), ≥ 98% identity to the corresponding germline sequence was considered as an unmutated IGHV gene.

### Statistical analysis

Kaplan-Meier curves were used to show the overall survival and the log-rank test was used to compare the survival between the groups. To calculate hazard ratios (HR) the Cox proportional hazard model (Cox-regression) was used. For all statistical analyses Stata v12.1 was used (StataCorp LP, College Station, TX, USA). P-values less than 0.05 were considered significant. Overall survival was measured from date of diagnosis until the last follow-up or death.

## Results and discussion

**Figure 1:**
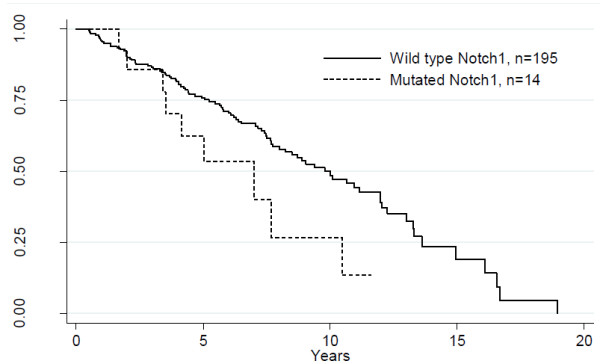
**Kaplan-Meier survival curves displaying a significant difference between patients with *NOTCH1 *mutation (n = 14) and *NOTCH1 *wildtype (n = 195) (p = 0.049).**

*NOTCH1* heterozygous mutations in the PEST domain occurred in a frequency of 14 out of 209 patients (6.7%) in our study. Thirteen of the mutations correspond to a 2-bp frameshift deletion, c.7541_7542delCT and one is a novel GT deletion at c.6988_6989delGT, both generating frameshift mutations, with subsequent stopcodon and truncated proteins eliminating the PEST domain. The Kaplan-Meier curve for the CLL-patients with *NOTCH1* mutations revealed a shorter overall survival (OS) compared to *NOTCH1* wildtype patients (p = 0.049) (Figure 1). Clinical and biological characteristics of the CLL patients in relation to the *NOTCH1* status are summarized in Table 2. Our study showed a similarity to other CLL studies, which have reported *NOTCH1* PEST domain mutations in 4.7% - 12.2% [6-11].

**Table 2:** Clinical and biological characteristics of the 209 CLL-patients

			***NOTCH1***				
	**Total**		**Wildtype**		**Mutated**		
	**n**	**%**	**n**	**%**	**n**	**%**	***P *value**
**Number of patients**	209		195		14		
**Sex**							0.67
male	145	69	136	70	9	64	
female	64	31	59	30	5	36	
**Binet stage**							0.11
A	101	48	98	50	3	21	
B/C	91	44	82	42	9	64	
Not determined	17	8	15	8	2	14	
**Treatment**							0.22
yes	182	87	168	86	14	100	
no	20	10	20	10	0	0	
Not determined	7	3	7	4	0	0	
**IGHV status**							0.22
Mutated	64	31	61	31	3	21	
Unmutated	123	59	113	58	10	71	
Not determined	22	10	21	11	1	7	
***TP 53 *status**							0.88
wild-type	196	94	183	94	13	93	
mutated	13	6	12	6	1	7	

T cell acute lymphoblastic leukemia (T-ALL) display high frequency of activating *NOTCH1* mutations including the extracellular heterodimerisation domain, in addition to elimination C-terminal PEST region mutations [21]. Studies in head and neck cancer have also identified mutations in the extracellular epiderminal growth factor repeat domain in the *NOTCH1* gene [22]. These studies prompted us to screen for mutations in those parts of the *NOTCH1* gene, but no mutations could be detected. NOTCH2 has a role in marginal zone B cell fate decision, similar to the critical role for NOTCH1 in determing the T-cell fate [13]. *NOTCH2* mutations are found in the C-terminal part close to the PEST region in splenic marginal zone lymphoma [23,24], but we did not find any mutations in the PEST region or heterodimerisation domain of the *NOTCH2* gene in our cohort.

**Figure 2:**
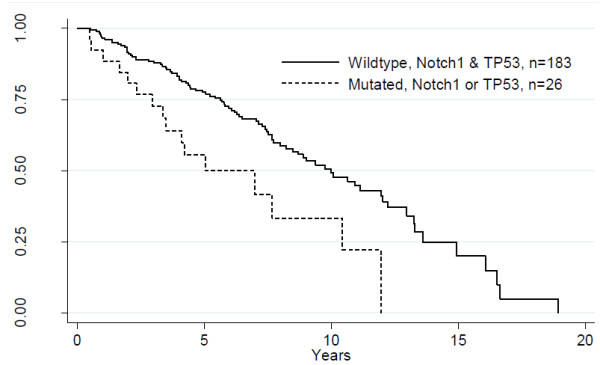
**A significant difference in overall survival between patients with *NOTCH1 *or *TP53 *mutations (n = 26) and CLL with non-mutated *NOTCH1 *and *TP53 *(n = 183*) *(p = 0.002).**

Mutations in the *TP53* gene are associated with poor prognosis in CLL [25]. Mutations in the *NOTCH1* gene are almost mutually exclusive with mutations in the *TP53* gene with or without 17p or 11q deletions, only one sample harbored mutations in both *NOTCH1* and *TP53*. Combined, *NOTCH1* (6.7%) and *TP53* (6.2%) mutations represent 12.9% of the patients in this cohort and indicated a significant worse survival as compared to wildtype *NOTCH1* and *TP53* (Log rank analysis, p = 0.002) (Figure 2). *NOTCH1* mutations may appear together with trisomy 12 [26,27], and in our cohort, trisomy 12 was found in 15/152 patients by BAC (bacterial artificial chromosome) microarray analysis and two of these carried *NOTCH1* mutation; this association was not significant (p = 0.56). By univariate analysis, the HR for death increased to 2.27 (1.32-3.91; 95% confidence interval) for tumors mutated in *NOTCH1* or *TP53* compared to *NOTCH1* and *TP53* wildtype tumors (p = 0.003) (Table 3). At the molecular level there seems to be an intriguing and complex link between p53 and NOTCH1. P53 induce NOTCH1 expression and seems to initiate an anti-apoptotic feedback mechanism with subsequent increased cell survival that may limit p53 promoting therapy with e.g. nutlins [28,29]. NOTCH signaling blockade by γ-secretase inhibitors to stimulate apoptosis may be considered to be of therapeutic value at least for wt p53 CLL patients [28].

**Table 3:** Analysis for overall survival

	**N**	**HR**	**95% CI**	***P***	**HR**^**1**^	**95%CI**	***P***
Wildtype *NOTCH1* and *TP53*	183	1			1		
Mutated *NOTCH1* or *TP53*	26	2.27	1.32-3.91	0.003	2.16	1.25-3.72	0.006
Wildtype *NOTCH1*	195	1			1		
Mutated *NOTCH1*	14	2.04	0.98-4.25	0.056	1.80	0.86-3.76	0.12
Wildtype *TP53*	196	1			1		
Mutated *TP53*	13	2.54	1.17-5.54	0.019	2.54	1.17-5.53	0.002

*HR*, hazard ratio; *CI*, confidence interval.

^1^Adjusted for age and sex.

**Figure 3:**
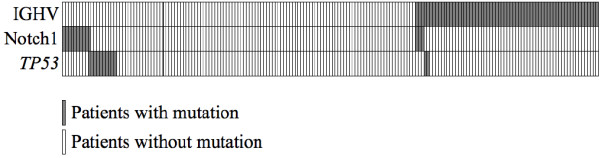
**Distribution of *NOTCH1 *mutations among IGHV gene status and *TP53 *gene status.** Ten of fourteen cases with mutated *NOTCH1* gene are found in the unmutated IGHV gene group and only one patient has both *NOTCH1* mutation and *TP53* mutation.

Among CLL patients with a mutated *NOTCH1* gene 10/14 (71%) had an unmutated IGHV gene in contrast to 113/195 (58%) with a wildtype *NOTCH1* gene, a difference that did not reach statistical significance (p = 0.22) (Figure 3 and Table 2). CLL with *NOTCH1* mutations seemed to be more progressive, with a high frequency of unmutated IGHV gene and advanced Binet stages, indicating a more aggressive disease course.

Five patients in this cohort had *NOTCH1* mutation at the time of diagnosis. For these patients the median time to first treatment was 101 days (range 21 to 145 days). For the whole group the median time from diagnosis to the first treatment was 438 days (range 0–6021 days). Further and expected, all patients with *NOTCH1* mutations identified at diagnosis had the more advanced Binet stages B and C, a tendency that due to few observations did not reach significance (p = 0.11) (Table 2). The frequency of *NOTCH1* mutations is also reported to be significantly higher in Richter syndrome, i.e., a progression of CLL into diffuse large lymphoma with often dismal outcome [8,30], however our cohort contained no information on the prevalence of Richter syndrome.

It is now recommended to perform *TP53* mutation analysis in patients with CLL as *TP53* mutations occur in about 5% of cases in absence of 17p deletion and represent an independent prognostic factor associated with worse outcome [31]. CLL patients with 17p deletion and/or *TP53* mutations are strongly associated with refractory disease, and also activated *NOTCH1* mutations were recently suggested to cause refractoriness to fludarabine [8,9,32].

## Conclusions

Our study confirms other recent reports that *NOTCH1* mutation eliminating the PEST domain, has a prognostic value as a novel risk marker in CLL similar to *TP53* mutations. Thus both *NOTCH1* and *TP53* mutation may be an indication for earlier and more active treatment or as an indicator for transplantation therapy.

## Competing interests

The authors declare that they have no competing interests.

## Authors’ contributions

KW collected data, performed experiments, analyzed and interpreted data, wrote the manuscript; RKD performed experiments, analyzed and interpreted data; JU performed experiments, analyzed and interpreted data; RG performed experiments and interpreted data; GJ interpreted data; MF performed statistical analysis; ML designed experiments; PS designed experiments, wrote the manuscript. All authors were involved in writing the manuscript. All authors read and approved the final manuscript.

## Pre-publication history

The pre-publication history for this paper can be accessed here:

http://www.biomedcentral.com/1471-2407/13/274/prepub

## References

[B1] Chiorazzi N Rai KR Ferranini M Chronic lymphocytic leukemia N Engl J Med 2005 352 805 815 10.1056/NEJMra041720 15728813

[B2] Wang L Lawrence MS Wan Y Stojanov P Sougnez C Stevenson K Werner L Sivachenko A DeLuca DS Zhang L Zhang W Zhang W Vartanov AR Fernandes SM Goldstein NR Folco EG Cibulskis K Tesar B Sievers QL Shefler E Gabriel S Hacohen N Reed R Meyerson M Golub TR Lander ES Neuberg D Brown JR Getz G Wu CJ SF3B1 and other novel cancer genes in chronic lymphocytic leukemia N Engl J Med 2011 365 2497 2506 22150006 10.1056/NEJMoa1109016 PMC3685413

[B3] Damle RN Wasil T Fais F Ghiotto F Valetto A Allen SL Buchbinder A Budman D Dittmar K Kolitz J Lichtman SM Schulman P Vinciguerra VP Rai KR Ferrarini M Chiorazzi N IgV gene mutation status and CD 38 expression as novel prognostic indicators in chronic lymphocytic leukemia Blood 1999 94 1840 1847 10477712

[B4] Hamblin TJ Davis Z Gardiner A Oscier DG Stevenson FK Unmutated IgV(H) genes are associated with a more aggressive form of chronic lymphocytic leukemia Blood 1999 94 1848 1854 10477713

[B5] Döhner H Stielgenbauer S Benner A Leupolt E Kröber A Bullinger L Döhner K Bentz M Lichter P Genomic aberrations and survival in chronic lymphocytic leukemia N Engl J Med 2000 343 1910 1916 11136261 10.1056/NEJM200012283432602

[B6] Sportoletti P Baldoni S Cavalli L Del Papa B Bonifacio E Ciurnelli R Bell AS Di Tommaso A Rosati E Crescenzi B Mecucci C Screpanti I Marconi P Martelli MF Di Ianni M Falzetti F Notch1 PEST domain mutation is an adverse prognostic factor in B-CLL British Journal of Haematology 2010 151 404 406 20813007 10.1111/j.1365-2141.2010.08368.x

[B7] Puente XS Pinyol M Quesada V Conde L Ordonez GR Villamor N Escaramis G Jares P Beà S González-Díaz M Bassaganyas L Baumann T Juan M López-Guerra M Colomer D Tubío JM López C Navarro A Tornador C Aymerich M Rozman M Hernández JM Puente DA Freije JM Velasco G Gutiérrez-Fernández A Costa D Carrió A Guijarro S Enjuanes A Whole genome sequencing identifies recurrent mutations in chronic lymphocytic leukemia Nature 2011 475 101 105 21642962 10.1038/nature10113 PMC3322590

[B8] Fabbri G Rasi S Rossi D Trifonov V Khiabanian H Ma J Grunn A Fangazio M Capello D Monti S Cresta S Gargiulo E Forconi F Guarini A Arcaini L Paulli M Laurenti L Larocca LM Marasca R Gattei V Oscier D Bertoni F Mullighan CG Foá R Pasqualucci L Rabadan R Dalla-Favera R Gaidano G Analysis of the chronic lymphocytic leukemia coding genome: role of NOTCH1 mutational activation J Exp Med 2011 208 1389 1401 21670202 10.1084/jem.20110921 PMC3135373

[B9] Rossi D Rasi S Fabbri G Spina V Fangazio M Forconi F Marasca R Laurenti L Bruscaggin A Cerri M Monti S Cresta S Famà R De Paoli L Bulian P Gattei V Guarini A Deaglio S Capello D Rabadan R Pasqualucci L Dalla-Favera R Foà R Gaidano G Mutations of Notch1 are an independent predictor of survival in chronic lymphocytic leukemia Blood 2012 119 521 529 22077063 10.1182/blood-2011-09-379966 PMC3257017

[B10] Shedden K Li Y Quillette P Malek SN Characteristics of chronic lymphocytic leukemia with somatically acquired mutations in exon 34 Leukemia 2011 26 1108 1110 22182918 10.1038/leu.2011.361 PMC3634572

[B11] Mansouri L Cahill N Gunnarsson R Smedby KE Tjönnfjord E Hjalgrim H Juliusson G Geisler C Rosenquist R NOTCH1 and SF3B1 mutations can be added to the hierarchical prognostic classification in chronic lymphocytic leukemia Leukemia 2013 27 512 514 23138133 10.1038/leu.2012.307

[B12] Rosati E Sabatini R Rampino G Tabilio A Di Ianni M Fettucciari K Bartoli A Coaccioli S Screpanti I Marconi P Constitutively activated Notch signaling is involved in survival and apoptosis resistance of B-CLL cells Blood 2009 113 856 865 18796623 10.1182/blood-2008-02-139725

[B13] Radtke F Wilson A Mancini SJC Robson MacDonald H Notch regulation of lymphocyte development and function Nature Immunology 2004 5 247 253 14985712 10.1038/ni1045

[B14] Palomero T Lim WK Odom DT Sulis ML Real PJ Margolin A Barnes KC O’Neil J Neuberg D Weng AP Aster JC Sigaux F Soulier J Look AT Young RA Califano A Ferrando AA Notch1 directly regulates c-MYC and activates a feed-forward-loop transcriptional network promoting leukemic cell growth PNAS 2006 103 18261 18266 17114293 10.1073/pnas.0606108103 PMC1838740

[B15] Lee SY Kumano K Nakazaki K Sanada M Matsumoto A Yamamoto G Nannya Y Suzuki R Ota S Ota Y Izutsu K Sakata-Yanagimoto M Hangaishi A Yagita H Fukayama M Seto M Kurokawa M Ogawa S Chiba S Gain-of-function mutations and copy number increases of Notch2 diffuse large B-cell lymphoma Cancer Science 2009 100 920 926 19445024 10.1111/j.1349-7006.2009.01130.x PMC11158873

[B16] Hubmann R Schwarzmeier JD Sheta M Hilgarth M Duechler M Dettke M Berger R Notch2 is involved in overexperssion of CD23 in B-cell chronic lymphocytic leukemia Blood 2002 99 3742 3747 11986231 10.1182/blood.v99.10.3742

[B17] Willander K Ungerbäck J Karlsson K Fredrikson M Söderkvist P Linderholm M MDM2 SNP309 promoter polymorphism, an independent prognostic factor in chronic lymphocytic leukemia European Journal of Haematology 2010 85 251 256 20491880 10.1111/j.1600-0609.2010.01470.x

[B18] Hallek M Cheson BD Catovsky D Caligaris-Cappio F Dighiero G Döhner H Hillmen P Keating MJ Montserrat E Rai KR Kipps TJ Guidelines for diagnosis and treatment of chronic lymphocytic leukemia: a report from an international workshop on chronic lymphocytic leukemia updating the national cancer institute working group 1996 guidelines Blood 2008 111 5446 5456 18216293 10.1182/blood-2007-06-093906 PMC2972576

[B19] Orita M Suzuki Y Sekiya T Hayashi K Rapid and sensitive detection of point mutations and DNA polymorphism using the polymerase chain reaction Genomics 1989 5 874 879 2687159 10.1016/0888-7543(89)90129-8

[B20] van Dongen JJ Langerak AW Brüggemann M Evans PA Hummel M Lavender FL Delabesse E Davi F Schuuring E García-Sanz R van Krieken JH Droese J González D Bastard C White HE Spaargaren M González M Parreira A Smith JL Morgan GJ Kneba M Macintyre EA Design and standardization of PCR primers and protocols for detection of clonal immunoglobulin and T-cell receptor gene recombinations in suspect lymphoproliferations: report of BIOMED-2 Concerted Action BMH4-CT98-3936 Leukemia 2003 17 2257 2317 14671650 10.1038/sj.leu.2403202

[B21] Weng AP Ferrando AA Lee W Morris JP 4th Silverman LB Sanchez-Irizarry C Blacklow SC Look AT Aster JC Activating mutations of NOTCH1 in human T cell acute lymphoblastic leukemia Science 2004 306 269 271 15472075 10.1126/science.1102160

[B22] Stransky N Egloff AM Tward AD Kostic AD Cibulskis K Sivachenko A Kryukov GV Lawrence MS Sougnez C McKenna A Shefler E Ramos AH Stojanov P Carter SL Voet D Cortés ML Auclair D Berger MF Saksena G Guiducci C Onofrio RC Parkin M Romkes M Weissfeld JL Seethala RR Wang L Rangel-Escareño C Fernandez-Lopez JC Hidalgo-Miranda A Melendez-Zajgla J The mutational landscape of head and neck squamous cell carcinoma Science 2011 333 1157 1160 21798893 10.1126/science.1208130 PMC3415217

[B23] Rossi D Trifonov V Fangazio M Bruscaggin A Rasi S Spina V Monti S Vaisitti T Arruga F Famà R Ciardullo C Greco M Cresta S Piranda D Holmes A Fabbri G Messina M Rinaldi A Wang J Agostinelli C Piccaluga PP Lucioni M Tabbò F Serra R Franceschetti S Deambrogi C Daniele G Gattei V Marasca R The coding genome of splenic marginal zone lymphoma: activation of NOTCH2 and other pathways regulating marginal zone development J Exp Med 2012 209 1537 1551 22891273 10.1084/jem.20120904 PMC3428941

[B24] Kiel MJ Velusamy T Betz BL Zhao L Weigelin HG Chiang MY Huebner-Chan DR Bailey NG Yang DT Bhagat G Miranda RN Bahler DW Medeiros LJ Lim MS Elenitoba-Johnson KS Whole-genome sequencing identifies recurrent somatic NOTCH2 mutations in splenic marginal zone lymphoma J Exp Med 2012 209 1553 1565 22891276 10.1084/jem.20120910 PMC3428949

[B25] Zenz T Kröber A Scherer K Häbe S Bühler A Benner A Denzel T Winkler D Edelmann J Schwänen C Döhner H Stilgenbauer S Monoallelic TP53 inactivation is associated with poor prognosis in chronic lymphocytic leukemia: results from a detailed genetic characterization with long-term follow-up Blood 2008 112 3322 3329 18689542 10.1182/blood-2008-04-154070

[B26] Baletti V Bottoni A Palamarchuk A Alder H Rassenti LZ Kipps TJ Notch1 mutations in CLL associated with trisomy 12 Blood 2012 119 329 331 22086416 10.1182/blood-2011-10-386144 PMC3257004

[B27] Del Guidice I Rossi D Chiaretti S Marinelli M Tavolaro S Gabrielli S Laurenti L Marasca R Rasi S Fangazio M Guarini A Gaidano G Foà R Notch1 mutations in +12 chronic lymphocytic leukemia (CLL) confer and unfavorable prognosis, induce a distinctive transcriptional profiling and refine the intermediate prognosis of +12 CLL Haematologica 2012 97 437 441 22207691 10.3324/haematol.2011.060129 PMC3291600

[B28] Secchiero P Melloni E di Iasio MG Tiribelli M Rimondi E Corallini F Gattei V Zauli G Nutlin-3 up-regulates the expression of Notch1 in both myeloid and lymphoid leukemic cells, as part of negative feedback antiapoptotic mechanism Blood 2009 113 4300 4308 19190243 10.1182/blood-2008-11-187708

[B29] Paolo Dotto G Crosstalk of Notch with p53 and p63 in cancer growth control Nature Rev. Cancer 2009 9 587 595 19609265 10.1038/nrc2675 PMC6059364

[B30] Rossi D Rasi S Spina V Fangazio M Monti S Greco M Ciardullo C Famà R Cresta S Bruscaggin A Laurenti L Martini M Musto P Forconi F Marasca R Larocca LM Foà R Gaidano G Different impact of NOTCH1 and SF3B1 mutations on the risk of chronic lymphocytic leukemia transformation to Richter syndrome British Journal of Haematoloy 2012 158 426 229 10.1111/j.1365-2141.2012.09155.x 22571487

[B31] Pospisilova S Gonzalez D Malcikova J Trbusek M Rossi D Kater AP Cymbalista F Eichhorst B Hallek M Döhner H Hillmen P van Oers M Gribben J Ghia P Montserrat E Stilgenbauer S Zenz T ERIC recommendations on *TP53* mutation analysis in chronic lymphocytic leukemia Leukemia 2012 26 1458 1461 22297721 10.1038/leu.2012.25

[B32] Zenz T Gribben JG Hallek M Döhner H Keating MJ Stielgenbauer S Risk categories and refractory CLL in era of chemoimmunotherapy Blood 2012 119 4101 4107 22394601 10.1182/blood-2011-11-312421 PMC4968336

